# Selective Soxhlets extraction to enhance solubility of newly-synthesized poly(indoloindole-selenophene vinylene selenophene) donor for photovoltaic applications

**DOI:** 10.1186/s40580-020-0219-9

**Published:** 2020-03-10

**Authors:** Jihyun Lim, Na Yeong Kim, Woongsik Jang, Un Su An, Aung Ko Ko Kyaw, Yun-Hi Kim, Dong Hwan Wang

**Affiliations:** 1 School of Integrative Engineering, Chung-Ang University, 84 Heukseok-Ro, Dongjak-gu, Seoul, 156-756 Republic of Korea; 2 Department of Chemistry and RINS, Gyeongsang National University, Jin-ju, 660-701 Republic of Korea; 3 Department of Electrical and Electronic Engineering, Southern University of Science and Technology, Shenzhen, 518055 People’s Republic of China

**Keywords:** Indoloindole, Donor polymer, Soxhlet, Additive, Organic solar cells

## Abstract

An electron-rich fused indoloindole-based poly(indoloindole-selenophene vinylene selenophene) was synthesized and characterized. Soxhlet can be obtained by continuously purifying the product with a specific solvent and obtaining a pure polymer with a high concentration. Molecular weight is affected by the vapor pressure of marginal solvent, and the polymer was fractionated using tetrahydrofuran, chloroform, and chlorobenzene. Solubility is closely related to the morphology of bulk heterojunction and device parameters. In the solution process of fabricating the organic solar cell, securement of solubility has a great effect on the performance of the device, because morphology and orientation of a photo-active layer which significantly affect charge transport in the device. Since tetrahydrofuran (THF) Soxhlet solvents have high vapor pressure and appropriate solubility parameters, THF induced the best solubility of P-IDI-SVS materials for organic solvents. And through additive optimization, the performance of the device based on P-IDI-SVS from THF-Soxhlet extraction was enhanced. This is expected to be a meaningful study because the effect on solubility of Soxhlet solvent suggests factors to be considered in the solution process in organic solar cell research. In addition, surface modified bulk heterojunction was observed using atomic force microscopy, photoluminescence, time-correlated single photon counting and Raman spectroscopy analysis.

## Introduction

Organic solar cells (OSCs) can be utilized in various devices based on their light and flexible characteristics [1], and they allow for the fabrication of large devices with low temperature and solution processes [2, 3]. It is also possible to tune the OSCs band gap through molecular control [4, 5]. With these advantages in mind, a variety of research has been conducted on OSCs using different approaches. However, the power conversion efficiency of OSCs still requires improvement. Thus, many research efforts have been made toward optimizing OSCs. Generally, the device interlayer is changed to increase performance, and heat treatment is performed to optimize the interface area.

In a bulk heterojunction (BHJ) solar cell, the absorption layer is composed of nanoscale-blended donor and acceptor molecules [3, 6]. Fullerene derivatives are often used as acceptors because the donor is usually conjugated with polymers, oligomers, or conjugated pigments. Therefore, properties of the donor–acceptor, such as energy level arrangement and charge carrier mobility, can be modified and designed by introducing new donors to optimize the OSCs [5, 7]. Dihydroindoloindole (IDI) derivatives have electron-rich, aromatic fused structures with two nitrogen atoms. Furthermore, they can be easily substituted with alkyl chains for better polymer solubility. Recently, our group developed new indoloindole-based donor–acceptor type donor polymers for OSCs. Polymer molecular weight typically also has a significant influence on the formation and morphology of resulting thin films [8]. Thus, it is a worthwhile approach to control solubility by differing the molecular weight without changing the molecular structure [9].

The Soxhlet extraction is a method for controlling polymer solubility via a post-synthesis step. The Soxhlet extraction is utilized for the extraction of a target material from a solid material containing impurities using a solvent in which the target is soluble and the impurities are insoluble. Therefore, the Soxhlet extraction method with various solvents can control polymer molecular weight by utilizing the solubility differences between the polymer and impurities for each solvent due to enrichment in a limited solubility.

**Scheme 1:**
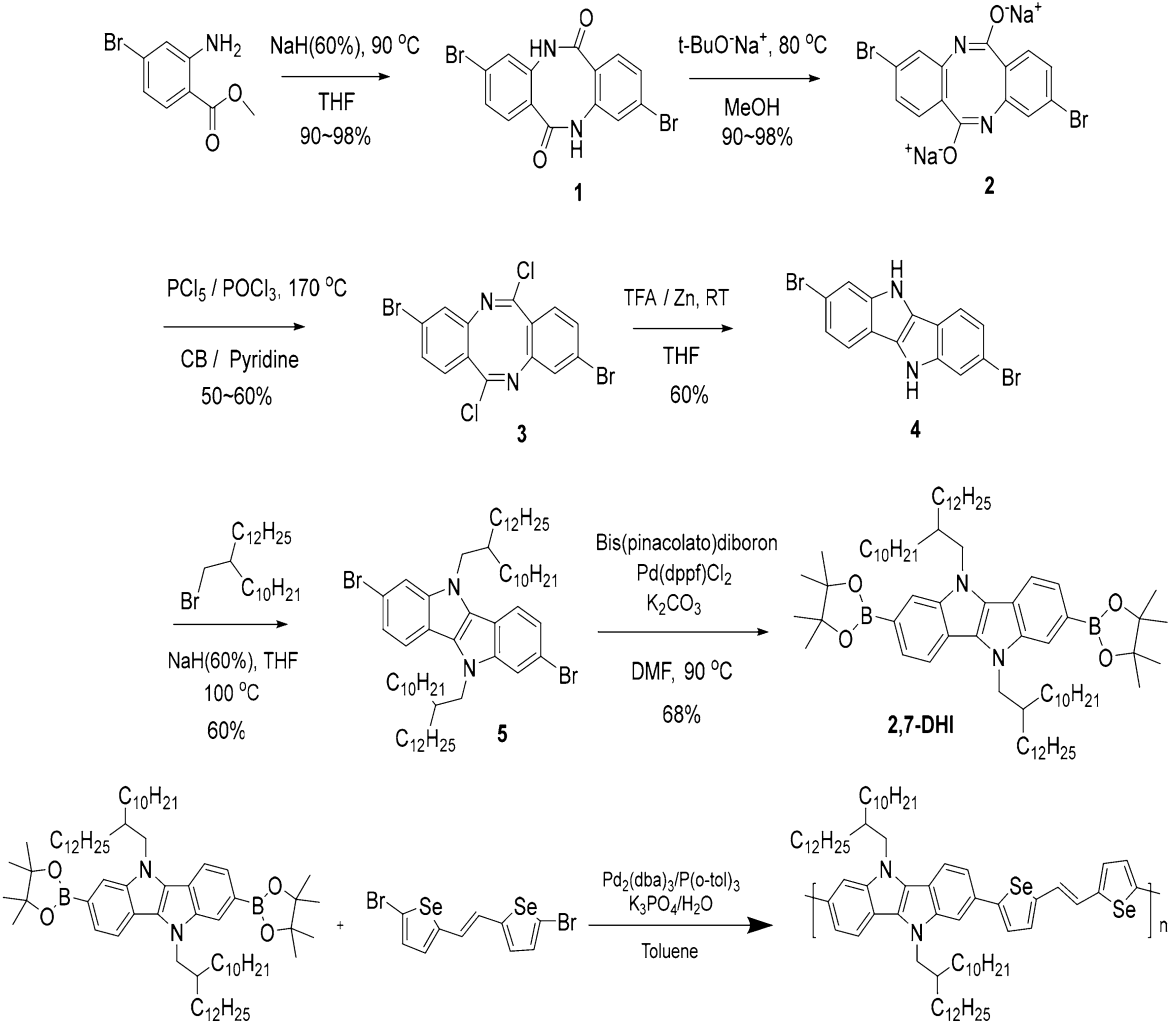
Synthesis of the P-IDI-SVS

In this work, we designed and synthesized the electron-rich donor–donor type compound poly(IDI-selenophene–vinylene–selenophene) (P-IDI-SVS, Scheme 1) by introducing SVS as a donor unit for organic electronics. After the synthesis, we obtained a series of P-IDI-SVS polymers through Soxhlet extraction using three different solvents: chlorobenzene (CB), chloroform (CF), and tetrahydrofuran (THF). Soxhlet is one of the commonly used techniques for solvent extraction of solid materials, which removes not only impurities, but also insoluble polymer fractions. The final synthesized polymer is placed in the thimble holder, slowly condensed and filled with fresh extraction solvent, and when the solution reaches the overflow level, the solution returns to the distillation flask below. Then it is repeated until extraction is completed and pure solid polymer without impurities is derived [10]. Even though the importance of Soxhlet process, there are few previous studies on the molecular weight changes after Soxhlet extraction. This paper describes the effect of Soxhlet on the tendency of solubility differences with marginal solvents. The results of Soxhlet extraction solvent were analyzed by investigating the blend film morphologies and device characteristics [11]. To improve the P-IDI-SVS BHJ morphology, we focused on the solubility of the polymers by applying additives. Consequently, we fabricated efficient BHJ solar cells based on the newly designed P-IDI-SVS donor polymer through solvent engineering. To demonstrate the solvent engineering effects, atomic force microscope (AFM), photoluminescence (PL), and time-correlated single-photon counting (TCSPC) measurements were conducted depending on the different Soxhlet solvent and type of additive.

## Experimental

### Materials

All starting materials were purchased from Sigma-Aldrich or Tokyo Chemical Industry. All solvents were purified by passage under N_2_ atmosphere. 5,10-Bis(2-decyltetradecyl)-2,7-bis(4,4,5,5-tetramethyl-1,3,2-dioxaborolan-2-yl)-5,10-dihydroindolo[3,2-b]indole and (E)-1,2-bis(5-bromoselenophen-2-yl)ethene were synthesized via published literature procedures [12]. Material used as the hole transport layer (HTL) was supplied by CLEVIOS™ with poly (3,4-ethylenedioxythiophene):polystyrenesulfonate (PEDOT:PSS), AI4083. Solvent of CB (chlorobenzene), CN (1-chloronaphthalene), DIO (1, 8-diiodooctane) and IPA (2-propanol) was used in Sigma Aldrich. PC_70_BM was purchased from an EM Index company in Korea. Titanium (IV) isopropoxide was supplied by Sigma Aldrich.

#### Synthesis of (E)-1,2-bis(5-bromoselenophen-2-yl)ethene

Under N_2_, (E)-1,2-di(selenophen-2-yl)ethene (1 g, 3.495 mmol), *N*-bromosuccinimide (1.49 g, 8.388 mmol), and DMF (25 mL) were combined and stirred for 3 h at room temperature. The solution was then diluted with H_2_O and extracted with dichloromethane. The organic extract was dried over MgSO_4_ and evaporated. The crude product was column chromatographed with hexane as the eluent and then crystallized from ether and methanol. Greenish white crystal, yield: 1.21 g (78.06%), ^1^H NMR (CDCl_3_, 300 MHz): δ (ppm) = 7.155 (2H, d, J = 1.4 Hz), 6.899 (2H, d, J = 1.4 Hz), 6.774 (1H, s).

#### Synthesis of 3,9-dibromodibenzo[b,f][1,5]diazocine-6,12(5H,11H)-dione (1)

Methyl 2-amino-4-bromobenzoate (30 g, 198 mmol) was dissolved in anhydrous THF (800 mL). Then, NaH (12.8 g, 533 mmol, 60% dispersion in mineral oil) was slowly added at room temperature. Next, the solution was stirred at 90 °C for 12 h. The solution was cooled to 25 °C and then poured slowly into a 2 M HCl anhydrous solution and ice to produce a solid. The precipitated product was collected by filtration, washed with distilled water (250 mL), and washed with methanol (50 mL) to afford 3,9-dibromodibenzo[b,f][1,5]diazocine-6,12(5H,11H)-dione (1). White solid (crude), 1H NMR MR (DMSO-d_6_, 500 MHz): δ (ppm) = 167.90, 135.55, 134.25, 134.04, 131.06, 128.49, 120.42, 67.49, HRMS-EI^+^ (m/z): Found: [M]^+^ 393.8955; C_14_H_8_Br_2_N_2_O_2_ requires [M]^+^ 393.8953.

#### Synthesis of sodium (5E,11E)-3,9-dibromodibenzo[b,f][1,5]diazocine-6,12-bis(olate) (2)

Under N_2_, **1** (50 g, 126 mmol) was dissolved into methanol (500 mL). Sodium t-butoxide (26.7 g, 278 mmol) was added slowly to the solution at room temperature, and the solution was stirred at 80 °C for 12 h. Finally, the methanol was removed by evaporation and the precipitated product was collected by filtration and washed with Et_2_O (50 mL) to afford sodium (5E,11E)-3,9-dibromodibenzo[b,f][1,5]diazocine-6,12-bis(olate) (2). White solid (crude), ^1^H NMR (DMSO-d_6_, 300 MHz): δ (ppm) = 6.85 (4H, s), 6.77 (2H, s), ^13^C NMR (DMSO-d_6_, 500 MHz): δ (ppm) = 170.87, 149.59, 135.39, 128.99, 126.46, 123.92, 119.99.

#### Synthesis of (5E,11E)-3,9-dibromo-6,12-dichlorodibenzo[b,f][1,5]diazocine (3)

Under N_2_, dried **2** (20 g, 45.5 mmol) was dissolved into CB (500 mL). Phosphorus oxychloride (21 mL, 227.5 mmol) and phosphorus pentachloride (28.4 g, 136.5 mmol) were poured slowly to the solution. Then, the solution was added dropwise to anhydrous pyridine (1.8 mL, 22.7 mmol) at room temperature. The reaction mixture was stirred at 170 °C for 2 h. After cooling to room temperature, CB was removed by distillation. The mixture was cooled to room temperature and poured slowly over sodium hydroxide and ice to produce a solid, and then the reaction mixture was neutralized to ~ pH 9. The reaction mixture was extracted with ethyl acetate, and the organic layer was dried with anhydrous MgSO_4_ and the solvent removed. The crude product was purified with column chromatography with dichloromethane and hexane (1:1) and recrystallized with acetone. After drying, (5E,11E)-3,9-dibromo-6,12-dichlorodibenzo[b,f][1,5]diazocine (3) was obtained. White, yield: 11.8 g (60%), ^1^H NMR (DMSO-d_6_, 300 MHz): δ (ppm) = 7.50–7.43 (4H, m), 7.33–7.32 (2H, m), ^13^C NMR (DMSO-d_6_, 500 MHz): δ (ppm) = 156.30, 146.06, 129.63, 129.40, 125.97, 124.79, 124.74, HRMS-EI^+^ (m/z): Found: [M]^+^ 429.8276; C_14_H_6_Br_2_Cl_2_N_2_ requires [M]^+^ 429.8275.

#### Synthesis of 2,7-dibromo-5,10-dihydroindolo[3,2-b]indole (4)

Compound **3** (10 g, 23.1 mmol) was dissolved into purified THF (46 mL), and zinc powder (18.1 g, 227.2 mmol) was poured into the mixed solution. Then, trifluoroacetic acid (41 mL, 554.4 mmol) was added dropwise at room temperature. The mixture was stirred at room temperature for 12 h and filtered to remove the zinc powder. The reaction mixture was extracted with ethyl acetate, and the organic phase was dried with MgSO_4_ and concentrated under reduced pressure. The crude product was washed with methanol, affording 2,7-dibromo-5,10-dihydroindolo[3,2-b]indole (4). White solid, yield: 5.0 g (60%), ^1^H NMR (DMSO-d_6_, 300 MHz): δ (ppm) = 11.35 (2H, s), 7.71 (1H, s), 7.69 (1H, d, J = 1.92 Hz), 7.24 (1H, d, J = 1.70 Hz), 7.21 (1H, d, J = 1.70 Hz), ^13^C NMR (DMSO-d6, 500 MHz): δ (ppm) = 168.73, 136.46, 132.74, 130.98, 130.67, 128.73, 123.62, HRMS-EI^+^ (m/z): Found: [M]^+^ 361.9048; C_14_H_8_Br_2_N_2_ requires [M]^+^ 363.9034.

#### Synthesis of 2,7-dibromo-5,10-bis(2-decyltetradecyl)-5,10-dihydroindolo[3,2-b]indole (5)

Compound **4** (5 g, 13.7 mmol) was dissolved into purified THF (300 mL) under N_2_. Then, NaH (2.0 g, 82.2 mmol, 60% dispersion in mineral oil) was slowly added, and the reaction mixture was stirred at 70 °C for 30 min at atmosphere. 11-(Bromomethyl)tricosane (17.2 g, 41.1 mmol) was added to the reaction mixture and stirred at 100 °C for 24 h. After completion, the reaction was cooled to room temperature. The reaction mixture was extracted with ethyl acetate, and the organic phase was dried with MgSO_4_ and concentrated under reduced pressure. The mixed compound was purified by column chromatography with *n*-hexane and recrystallized with dichloromethane and acetone. After drying, 2,7-dibromo-5,10-bis(2-decyltetradecyl)-5,10-dihydroindolo[3,2-b]indole (5) was obtained. White, yield: 8.55 g (60%), ^1^H NMR (CD_2_Cl_2_, 300 MHZ): δ (ppm) = 7.73 (2H, d, J = 8.4 Hz), 7.63 (2H, d, J = 1.5 Hz), 7.28 (2H, dd, J = 1.5, 1.8 Hz), 4.34 (4H, d, J = 9 Hz), 2.19–2.16 (4H, br), 1.29–1.20 (80H, br), 0.91 (12H, t, J = 6.78, 6.78 Hz), ^13^C NMR (CDCl_3_, 500 MHz): δ (ppm) = 141.60, 126.12, 121.33, 118.78, 115.29, 113.16, 113.05, 50.02, 38.64, 31.93, 31.62, 29.91, 29.67, 29.61, 29.53, 29.39, 29.35, 26.45, 22.71, 14.14, HRMS-FAB^+^ (m/z): Found: [M]^+^ 1034.6669; C_62_H_104_Br_2_N_2_ requires [M]^+^ 1034.6566.

#### Synthesis of 2,7-DHI

Compound **5** (3.5 g, 3.37 mmol) was dissolved into anhydrous DMF (200 mL) under N_2_. Then, potassium acetate (4.3 g, 28.6 mmol), bis(pinacolato)diboron (4.2 g, 16.87 mmol), and Pd(dppf)_2_Cl_2_ (0.25 g, 0.34 mmol) were added, and the reaction mixture was stirred at 90 °C for 12 h. After completion, DMF was removed by distillation. The reactant was extracted with dichloromethane, and the organic phase was dried with MgSO_4_ and concentrated under reduced pressure. The mixture was purified by column chromatography (dichloromethane:hexane = 1:2) and recrystallized with dichloromethane and acetone. After drying, 2,7-DHI was obtained. White, yield: 2.6 g (68%), ^1^H NMR (CD_2_Cl_2_, 300 MHz): δ (ppm) = 7.94 (2H, s), 7.88 (2H, d, J = 8.1 Hz), 7.57 (2H, d, J = 8.1 Hz), 4.45 (1H, d, J = 7.50 Hz), 2.28–2.27 (2H, br), 1.14–1.20 (104H, br), 0.91 (12H, t, J = 6.76, 6.76 Hz), ^13^C NMR (CD_2_Cl_2_, 500 MHz): δ (ppm) = 140.99, 127.27, 123.75, 117.33, 116.60, 116.10, 83.51, 49.64, 38.77, 31.91, 31.57, 29.91, 29.66, 29.59, 29.52, 29.36, 29.33, 26.44, 24.72, 22.68, 22.68, 13.87, HRMS-FAB^+^ (m/z): Found: [M]^+^ 1131.0029; C_62_H_10_4Br_2_N_2_ requires [M]^+^ 1131.0060.

### Instrumentation

The ^1^H NMR spectra were recorded using a Bruker DRX 300 MHz spectrometer. Mass spectra were measured using a JEOL JMS-700. The thermal analysis measurements were performed using a thermogravimetric analyser (TGA) (TGA 2050, TA Instruments) under N_2_, and the samples were heated at 10 °C/min. Differential scanning calorimetry (DSC) was conducted under N_2_ using a TA Instruments 2100 DSC. The samples were heated at 10 °C/min from 0 to 350 °C. UV–vis absorption spectra were measured using a PerkinElmer LAMBDA-900 UV/vis/IR spectrophotometer. Cyclic voltammograms of materials were recorded on an Epsilon E3 at room temperature in a 0.1 M solution of tetrabutylammonium perchlorate (Bu_4_NClO_4_) in acetonitrile under N_2_ at a scan rate of 50 mV/s. A Pt wire was used as the counter electrode and an Ag/AgCl electrode was used as the reference electrode.

### Polymerization of P-IDI-SVS

The polymer was synthesized by the Suzuki coupling reaction using palladium catalysts. 5,10-Bis(2-decyltetradecyl)-2,7-bis(4,4,5,5-tetramethyl-1,3,2-dioxaborolan-2-yl)-5,10-dihydroindolo[3,2-b]indole (0.3 g, 0.265 mmol) and (E)-1,2-bis(5-bromoselenophen-2-yl)ethene (SVS-Br) (0.117 g, 0.265 mmol) were dissolved into dry toluene (8 mL) and nitrogen-bubbled for 20 min in a Schlenk flask. Then, Pd_2_(dba)_3_ (0.003 g, 0.0039 mmol), P-(o-tol)_3_ (0.007 g, 0.0238 mmol), K_3_PO_4_ (0.225 g, 1.060 mmol), distilled water (3 mL), and Aliquat 336 (1 drop) were added, and the reaction mixture was stirred at 95 °C under N_2_ for 80 h. Then, phenyl boronic acid was added and stirred into the reaction mixture for 6 h, and bromobenzene was added and stirred for 6 h for end capping. After cooling down, the mixture was precipitated in methanol. The filtered polymer was purified and fractionated by Soxhlet using methanol, acetone,* n*-hexane, THF, and CF. Each of the THF, CF, and CB fractions were evaporated under reduced pressure, and the product was precipitated in methanol, filtered, and finally dried under high vacuum. The Soxhlet portions of the polymer were fractionated to investigate the molecular weight effect [10]. The number-average molecular weights (Mn) of the polymers were estimated by gel permeation chromatography using a polystyrene standard with THF and CF solvent. The THF and CF Soxhlet portions had Mn = 109 kDa with 1.015 of PDI and Mn = 128 kDa with 1.006 of PDI. Yield: 0.24 g (85.71%), ^1^H NMR (CDCl_3_, 500 MHz): δ (ppm) = 8.20–6.28 (12H, br), 4.42–3.37 (6H, br), 1.38–0.81 (92H, br).

### Device fabrication

P-IDI-SVS:PC_70_BM organic solar cells based on bulk heterojunctions were fabricated on indium tin oxide (ITO) coated glass substrates. The glass substrate coated with ITO was washed with detergent and sonicated with distilled water, acetone and isopropyl alcohol (IPA) for 20 min. After washing, IPA is dried with nitrogen, placed in an oven and dried for more than 20 min. ITO was subjected to UV-ozone treatment for 15 min to change the surface properties of ITO from a hydrophobic surface to a hydrophilic surface. A hole transport layer of PEDOT:PSS (Al4083) was spin-coated on ITO (thickness: 40 nm or less). The PEDOT:PSS substrate was annealed in air at 140 °C for 10 min to remove the solvent. A P-IDI-SVS:PC_70_BM solution was prepared at a concentration of 20 mg/mL as a function of a donor:acceptor ratio (1:2 w/w) in CB with 1-chloronaphthalene (CN) 3 vol% and diiodooctane (DIO) 3 vol%. This solution was spin-casted on top of the PEDOT:PSS layer in an Ar-filled glove box after filtering. Then, a 10 nm or less TiOx layer as an electron transfer layer was formed on the photoactive layer by spin-coating. A 100 nm aluminium layer as a cathode electrode was then deposited under a pressure of 2.0 × 10^–6^ Torr through thermal evaporation. All devices were encapsulated using a resin and a cover glass.

### Characterization

The current density–voltage (J–V) characteristics and impedance spectroscopy of the OPV were measured using a ZIVE SP1 and solar simulator with illuminated AM 1.5 Global conditions at an intensity of 100 mW/cm^2^ and a cell area of 0.15 cm^2^. Short-circuit current verification of *J*_*SC*_ related to J–V curve by measuring incident photocurrent efficiency (IPCE) spectrum of solar cell after power calibration (ABET technologies, Inc., LS150, USA) using a monochromatic chromatograph (Dongwoo OPTRON Co., Ltd., MonoRa-500i, Korea). The surface morphology of the blend of P-IDI-SVS:PC_70_BM was observed by AFM (Park NX10) using non-contact mode. The PL spectra were measured by fluorescence spectroscopy (F-7000 fluorescence spectroscopy, HITACHI, Tokyo, Japan) for the quenching rate of the active layer of P-IDI-SVS:PC_70_BM. The excitation wavelength was 530 nm and the PMT voltage was 700 V. The time-correlated single photon counting (TCSPC) technique (XperRam Ultimate) under excitation from a 405 nm laser a 20 MHz repetition rate. The Raman Intensity were measured by Raman microscopy (Xperam200(Nanobase Inc.). The laser wavelength was 532 nm, and the power was 4 µW for each device. The magnification of the object lens was 50×. We calculated the average of ten datasets of Raman spectra in the same position for each sample.

## Results and discussion

The synthetic scheme of monomers and polymer is given in Scheme 1. The polymerization of P-IDI-SVS was performed with a Suzuki coupling reaction. The polymer was purified and fractionated by Soxhlet using methanol, acetone, *n*-hexane, THF, CF, and CB. P-IDI-SVS(CB) was extracted with CB, P-IDI-SVS(CF) with CF, and P-IDI-SVS(THF) with THF, respectively. In the Soxhlet extraction process, the extraction rate depends on the solvent evaporation rate associated with the difference in the vapor pressure of the solvent. The higher the vapor pressure, the faster the transition from liquid to gas at the same atmospheric pressure, so that the product can be extracted by rotating the solvent. As the solvent is rotated several times, only products except impurities are extracted. The circulation of the solvent proceeds repeatedly and the final polymer is dissolved sequentially. It extracts only pure polymers, excluding polymers that do not dissolve when applied to solution process and the impurity of the synthesized final polymer [10]. Therefore, since these polymers were extracted by different solvents, the polymers exhibited different solubility due to differences in concentration depending on the solvents. Soxhlet reaction rate is greatly affected by both vapor pressure and solubility of marginal solvent. The molecular weight according to the Soxhlet solvent differs greatly depending on the vapor pressure of the marginal solvent [9]. The vapor pressure of the marginal solvent is proportional to the speed of the Soxhlet extraction. This is because the faster the extraction speed, the faster the evaporation rate of the solvent. The vapor pressure of CB is 12 hPa, 210 hPa for CF, and 200 hPa for THF, respectively (at 20 °C) [13]. According to the vapor pressure, the vapor pressure is more than 15 times higher in CF and THF solvents than CB solvents. Also, the solubility parameter of Soxhlet marginal solvent plays an important role in dissolving the polymer. The role of the solubility parameter is to predict whether one material is dissolved in another to form a solution. We searched solubility parameter of each Soxhlet solvent. The parameters of IDI-SVS are expected to be more soluble in THF Soxhlet solvent than CF Soxhlet solvent [14]. The solubility parameter values of CF and THF are more than 9 δ and the value of THF is 9.6 δ, which is especially higher than the CF value (9.3 δ) [15, 16]. With these values, it was expected that the THF would be slightly smaller in molecular weight. In fact, the molecular weight of THF was measured to be lower than the molecular weight of CF (see “Experimental” section). As a result, the solubility test was performed according to the molecular weight of the Soxhlet solvent.

**Fig. 1:**
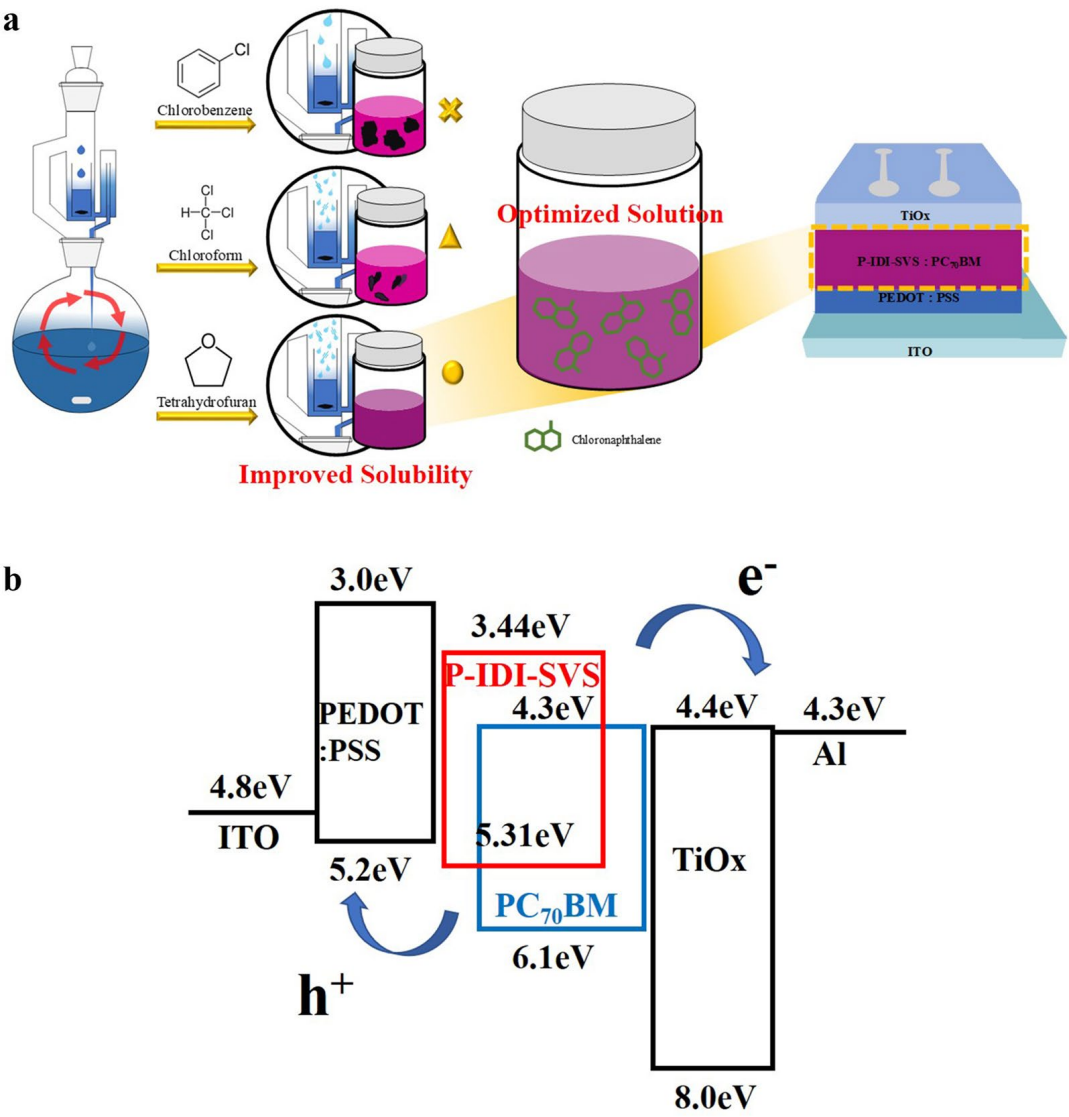
**a** Image of solubility change due to Soxhlet solvent and device structure of P-IDI-SVS:PC_70_BM BHJ solar cell. **b** Energy level diagram of the organic solar cell

Indeed, when each polymer was added to CB at 80 °C, P-IDI-SVS(CB) was almost insoluble, whereas P-IDI-SVS(THF) dissolved well (Additional file 1: Fig. S1). This result suggested that a P-IDI-SVS(THF) film could be formed with a uniform surface morphology, which would be advantageous for device fabrication (Fig. 1a). The structures of the synthesized monomers and copolymers were confirmed using various spectroscopies such as ^1^H NMR, ^13^C-NMR, and MS. The thermal stability of P-IDI-SVS(THF) was investigated with TGA and DSC under N_2_. As shown in Scheme 1-1, a 5% weight loss was observed at 420.19 °C. The polymer did not show any transitions in the DSC analysis. The results of TGA and DSC analyses indicate that the polymer has a good thermal stability. The UV–vis absorption spectra of P-IDI-SVS(THF) and as a spin-coated thin film are shown in Additional file 1: Fig. S2 UV–vis absorption maxima of P-IDI-SVS(THF) were observed at 556 and 602 nm, whereas the absorption maximum for the thin film occurred at 561 and 603 nm. In the film state, the shoulder peak at 603 nm was more intense than in the solution. The annealed film showed distinct peaks at 545 nm and 585 nm, and the polymer showed an extended conjugated structure. The optical band gap of the P-IDI-SVS was determined from the UV–vis absorption onsets in the solid state with the empirical equation (Eg = 1240/λ onset eV). And the optical band gap of the P-IDI-SVS was found to be 1.87 eV [17].

To observe the dependence of photovoltaic effects on the Soxhlet extraction solvent, we fabricated organic photovoltaics (OPVs) with structures of ITO/PEDOT:PSS/P-IDI-SVS(CB, CF, or THF):PC_70_BM/TiO_x_/Al (Fig. 1a). Figure 1a explains the overall experimental procedure of this paper, and explains the difference in solubility between P-IDI-SVS and Soxhlet solvent. The highest occupied molecular orbital and lowest unoccupied molecular orbital (HOMO and LUMO) energy levels of P-IDI-SVS were estimated as − 5.31 eV and − 3.44 eV (bandgap energy of 1.8 eV), respectively, as shown in Fig. 1b, according to the onset oxidation and reduction potentials from electrochemical cyclic voltammetry measurements (Additional file 1: Fig. S3). It is measured at a level similar to the optical band gap and is judged to be reliable. And the energy level diagram shows that the electrons are highly mobile, making the material suitable for use as a donor. When light enters the active layer, the electrons are in the excited state in the conduction band and the holes are in the valence band in the empty space after the electrons are excited. At this time, the LUMO level has a form in which electrons can move in a stepwise manner, and the HOMO level shows a stepped shape in which the hole can be moved to the electrode well, so that the movement of the electrons can be expected to be smooth [18].

P-IDI-SVS(THF) with the highest solubility was found to have a very low efficiency of 1.77% in CB solvent (Additional file 1: Table S1). Therefore, we tried to improve the solubility of the active layer and the morphology of the thin film by adding additives to the solvent. The effect of each additive is described as follows. The DIO and CN suppresses the volatility of the solvent due to its high boiling point and leads to slow evaporation of the solution. The slow evaporation can prevent the aggregation of the acceptor PC_70_BM to induce nano-phase separation, because PC_70_BM easily pulls each other [19]. Although the additives exhibit a similar effect, additive optimization is necessary because there is a suitable material for each additive. Therefore, we applied both DIO and CN to the device. Therefore, the DIO material, which is known as a representative material of the solvent additive, was applied as an additive to secure the solubility of the donor material with respect to the solvent [20]. Also, DIO additive suppresses all aggregates in the BHJ and significantly reduces the domain size, thereby improving the PCE by improving *J*_*SC*_ [20–22]. P-IDI-SVS(THF) was used as a donor to form an organic active layer, and the additive was applied to the solvent to optimize the device.

The P-IDI-SVS(CB) had a power conversion efficiency (PCE) of 2.14% due to its relatively low fill factor (FF) and short-circuit current density (*J*_*SC*_) when DIO is added to the solvent CB [23]. These properties resulted from poor film formation due to the lower solubility of the polymer, as shown in Additional file 1: Fig. S1. In addition, P-IDI-SVS(CF) showed better solubility (Additional file 1: Fig. S1) and improved efficiency to 2.25%, but there was a limit to optimization of efficiency. On the other hand, the most significant performance improvement was observed in P-IDI-SVS(THF) devices. The efficiency of P-IDI-SVS(THF) was improved to 3.15% when solubility was improved by control of appropriate solvent additives and extraction of Soxhlet from THF as compared to P-IDI-SVS(CB).

**Fig. 2:**
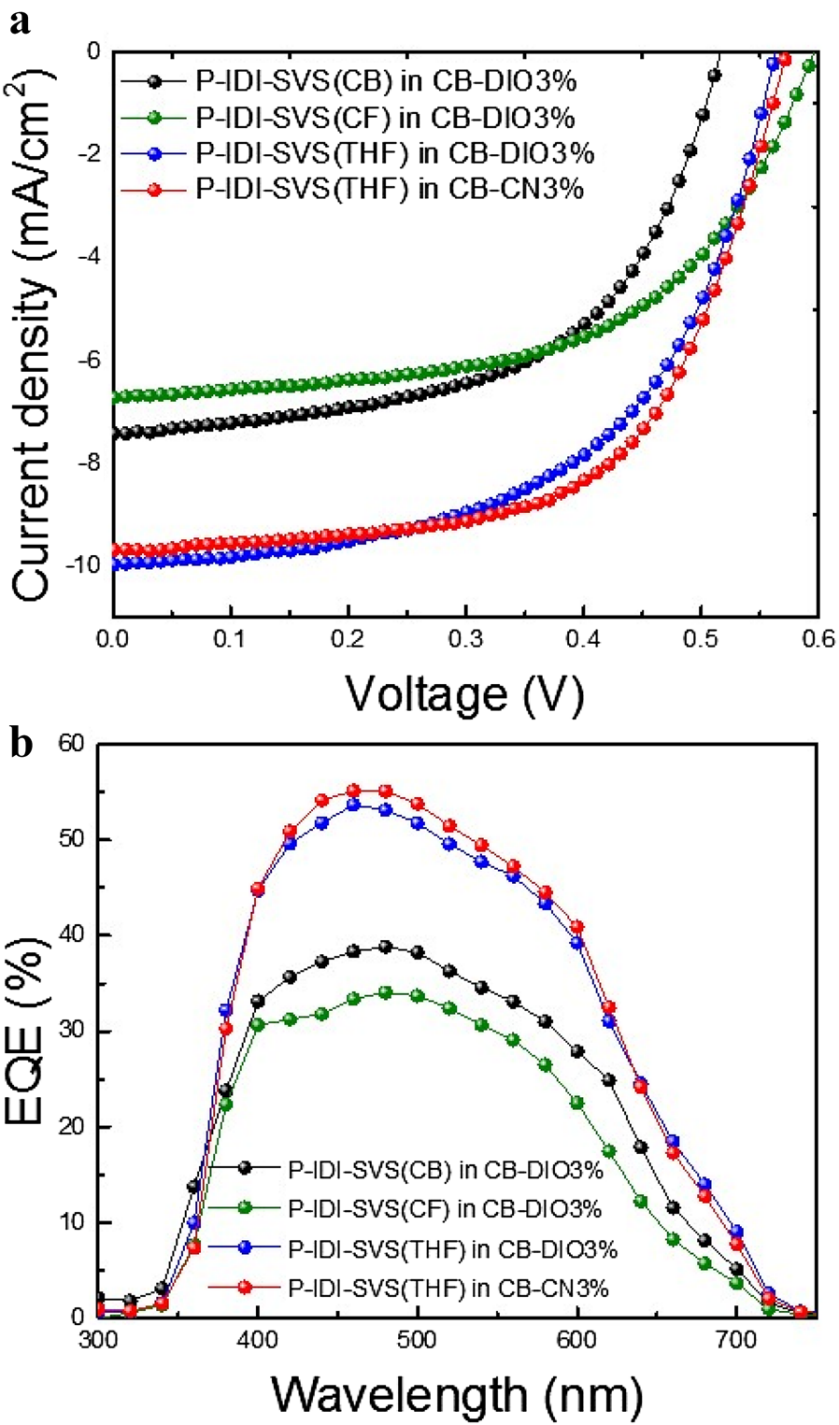
**a** J–V curves of different Soxhlet fraction P-IDI-SVS BHJ devices processed, **b** EQE of different Soxhlet P-IDI-SVS BHJ devices processed

To further optimize this P-IDI-SVS(THF), another additive, CN, was used. The use of CN additives reduces the evaporation rate of the solvent, which takes a long time for the self-organization of the polymer chains of BHJ to form phase separation, resulting in high hole mobility. It also forms a smoother and more uniform well-aligned network, providing efficient charge transport and FF [24–26]. When the CN additives were included in the BHJ blend, the performance of the P-IDI-SVS(THF) device improved to 3.38% with increased *J*_*SC*_ and FF compared to the DIO additive [27–29]. This is because the addition of CN to the CB solvent improved the surface modification of the BHJ by self-organization. The current density–voltage (J–V) characteristics of the OPV devices based on P-IDI-SVS(CB, CF, or THF):PC_70_BM BHJ in CB–DIO3% and CB–CN3% solvent are shown in Fig. 2, and detailed device characteristics are summarized in Additional file 1: Table S1.

**Fig. 3:**
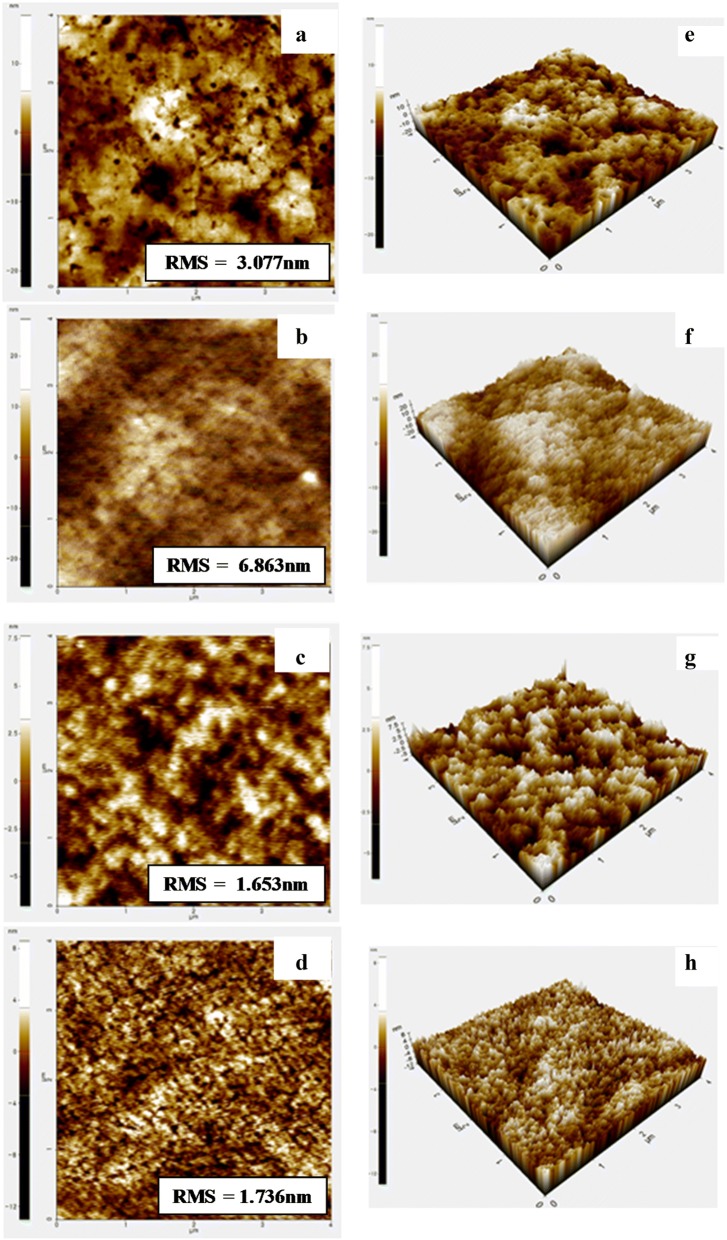
Non-contact mode AFM 2D height images of BHJ films based on **a**–**c** in CB–DIO3% solvent and **d** in CB–CN3% solvent, **a** P-IDI-SVS(CB):PC_70_BM, **b** P-IDI-SVS(CF):PC_70_BM, **c**, **d** P-IDI-SVS(THF):PC_70_BM. Non-contact mode AFM 3D height images of BHJ films based on based on **e**–**g** in CB–DIO3% solvent and **h** in CB–CN3% solvent, **e** P-IDI-SVS(CB):PC_70_BM, **f** P-IDI-SVS(CF):PC_70_BM, and **g**, **h** P-IDI-SVS (THF):PC_70_BM

The external quantum efficiency (EQE) spectrum of the device shown in Fig. 2b clearly showed its effect depending on the Soxhlet solvent. An increase of about 27% in THF solvent compared to Soxhlet CB solvent was found in the integrated *J*_*SC*_ form EQE. This indicates that P-IDI-SVS is extracted from the THF solvent, resulting in improved solubility and thus a significant improvement in EQE over the entire wavelength range [30, 31]. Furthermore, the EQE of the device using CN as an additive for optimization was slightly increased compared to CB–DIO3% solvent. It is observed that the use of the additive CN rather than the additive DIO in the solvent contributes to the increase of the current value by improving the charge flow. This phenomenon can be explained based on the morphology and roughness as seen in the AFM images in Fig. 3.

Atomic force microscope analysis was performed to observe the BHJ thin film morphology of the device according to the difference in solubility. Figure 3 shows the AFM measurements for thin films spin-coated at the same rate for each solution to observe the surfaces of the P-IDI-SVS(CB, CF, and THF). This is because the morphology of each layer plays an important role in determining the performance of device applications. The RMS value of P-IDI-SVS(THF) was the lowest, and the P-IDI-SVS(THF) film showed the lowest roughness and flatness. This indicates that P-IDI-SVS(THF), with the most improved solubility, formed more uniform film than P-IDI-SVS(CB and CF) [32]. Since the flat active layer can contribute to uniform formation of the electron transport layer, the device has improved FF and *J*_*SC*_ [28, 30]. Based on these results, the P-IDI-SVS(THF) material was optimized, and a solvent additive was used to improve the phase separation of the donor–acceptor [33]. The surface roughness and the RMS value of films with CB–CN3% and CB–DIO3% additives were the lowest values at approximately 1.6 ≈ 1.7 nm. This is because phase separation was optimized by controlling the evaporation rate of the solvent after the addition of the DIO solution, resulting in a more uniform film forming effect and improved *J*_*SC*_ and PCE (%) [29, 34, 35]. The additive CN also resulted in a more uniform thin film, leading to efficient charge transport [36]. In CB–CN3% and CB–DIO3%, the roughness of the thin film is reduced, and the charge carrier migration is improved, which enhances the *J*_*SC*_ and improves the performance of the photovoltaic device.

**Fig. 4:**
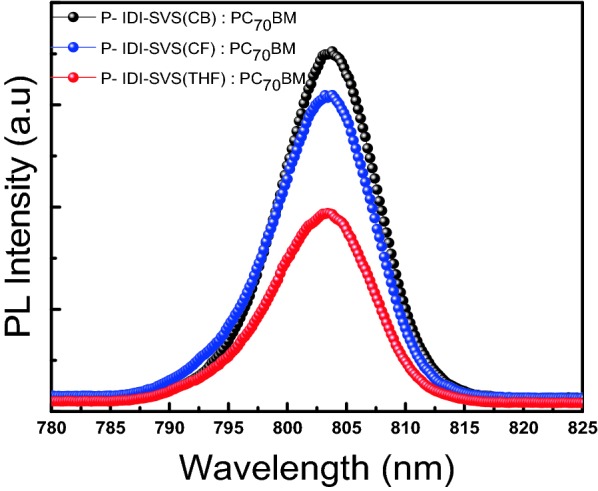
PL intensities of different Soxhlet fraction P-IDI-SVS:PC_70_BM BHJ devices processed from solutions with CB added CN3%

Figure 4 shows the intensity of photoluminescence (PL) in fixed solvents with different P-IDI-SVS(CB, CF and THF):PC_70_BM. PL intensities indicate the fastest drop of light energy from LUMO to HOMO, provides direct evidence of exciton dissociation and compares and analyses degree of recombination in the active layer. PL represents the intensity of light emitted from the active layer and then emitted back to the active layer, which is closely related to the charge generation and recombination of the active layer. The greater the amount of charge generated, the more charge is transferred, which increases the current flow in the device. This affects the increase of the efficiency of the device [37]. This means that the reduced the PL intensity, the recombination is inhibited, leading to faster the quenching occurs. Therefore, recombination of charges generated in the photoactive layer of the device in which CN is added to CB is suppressed. This is evidence that excitons are well separated from the BHJ layer, and this improved charge generation causes *J*_*SC*_ to rise and this leads to good device performance.

**Fig. 5:**
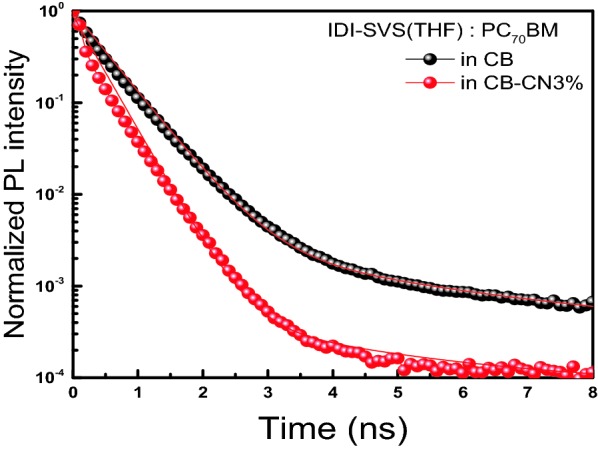
TCSPC results of P-IDI-SVS(THF):PC_70_BM measured with different solvents (CB, CB–CN3%)

Figure 5 show the result of time-correlated single photon counting (TCSPC) measurement. It is possible to measure the excitation life time by measuring the decay time of the PL. We measured the quenching rate by TCSPC analysis of P-IDI-SVS(THF):PC_70_BM film with CB–CN3% solvent to confirm the effect of additive. Among them, CB–CN3% showed an improved quenching rate of 350 ps (τ). On the other hand, the quenching rate was reduced to 503 ps (τ) in CB solvent (Additional file 1: Table S2). This indicates that when the solvent CB–CN3% is applied, the charge transport occurs well and the recombination rate of the BHJ excitons decreased [38]. Therefore, the active layer using the P-IDI-SVS(THF) donor increases the quenching rate at CB–CN3% via solvent additive, resulting in rapid exciton separation [39]. Since this is related to PL measurement data, a clearer basis can be obtained, and we can see that the control of the additives contributed to the PCE improvement as the *Jsc* increased.

**Fig. 6:**
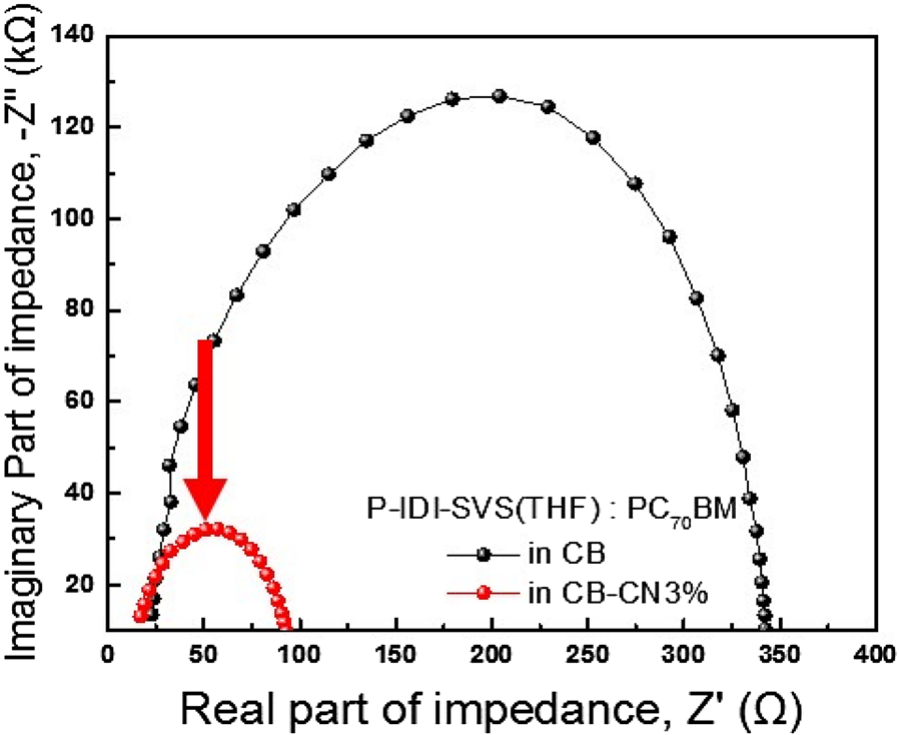
Impedance spectra of P-IDI-SVS(THF):PC_70_BM BHJ solar cell with different solvents (CB, CB–CN3%)

Figure 6 shows the measurement of the device at the impedance in a zero bias environment by comparing the additive applied to the solvent with P-IDI-SVS(THF). When the device in illumination is applied a zero bias, the internal voltage exhibits maximum voltage and minimum current, which means an open circuit condition. Since the almost charges of the device recombined in this state, the resistance at impedance spectroscopy represents the charge transport resistance [40]. As a result, the resistance of charge transport is reduced when additives are added, rather than using pure solvents. As a result, the resistance of charge transport is reduced when additives are added, rather than using pure solvents. Especially, when CN additive is used as a solvent for BHJ, charge transport resistance is lowest when CN additive is applied and therefore charge transfer is the most improved [41].

**Fig. 7:**
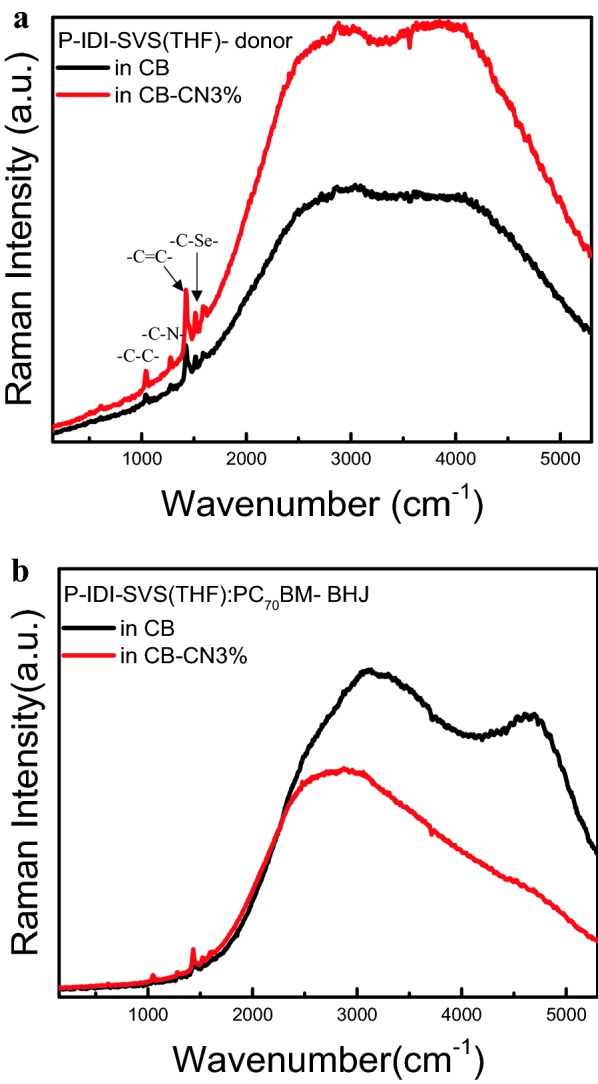
Raman Spectroscopy of **a** P-IDI-SVS(THF) (only donor) and **b** P-IDI-SVS(THF):PC_70_BM (BHJ films) of donor only films with CB and CB–CN3% solvent

Figure 7 shows the distribution of BHJ and only donor components in the device by Raman spectroscopy. In the BHJ spectrum, strong Raman peaks are observed at 1380 cm^−1^ and 1442 cm^−1^, which means skeletal stretching of C–C and stretching vibration of C=C, respectively. In addition, the C–N bond and the C–Se bond were observed in the Raman peaks in Fig. 7a, confirming that the qualities of P-IDI-SVS were well observed [42–44]. In the Raman graph of Fig. 7a, stronger intensities and clear peaks can be found in the only donor containing CN additives. The effect obtained by adding CN is to form a uniform and flat thin film. Fluorescent backgrounds are also observed in this spectrum. In the fluorescence background of Fig. 7a, the addition of the CN additive showed excellent photosensitization reaction. As shown in Fig. 7b, when the CN was added to the solvent, the fluorescence signal of BHJ decreased, compared with CB, overall charge generation is more likely to occur and quenching is more likely to occur on the long wavelength side, contributing to charge generation, which is observed at the same location, and in the fluorescent background spectrum. Thus, it is expected that more charge generation and current increase effects can be expected in the BHJ layer with more donor and acceptor blended in CB–CN3%.

## Conclusions

In summary, we newly synthesized P-IDI-SVS by introducing SVS as a donor unit for excellent intermolecular interactions. We also obtained purified P-IDI-SVS polymers via Soxhlet extraction using three different solvents: CB, CF, and THF, respectively. The molecular weight of P-IDI-SVS is determined during the Soxhlet extraction process and the molecular weight varies depending on the Soxhlet extraction solvent. This molecular weight of polymer is closely influenced by the vapor pressure and solubility parameter of the extraction solvent. The higher the vapor pressure, the faster the circulation of the Soxhlet extraction solvent, and the more suitable solubility, the better the dissolution of P-IDI-SVS and the easier extraction. After the Soxhlet extraction process, the molecular weight of the polymer is determined to have different solubilities. The Soxhlet solvent of THF showed low molecular weight based on excellent vapor pressure and solubility. AFM measurements showed a flat and uniform thin film with the best surface morphology of the samples. P-IDI-SVS(THF):PC_70_BM had the highest PCE (3.15%) due to its increased *J*_*SC*_. To improve the P-IDI-SVS BHJ, we also focused on the solubility of the polymers by applying another additive. As a result of this solvent engineering, we created an efficient P-IDI-SVS(THF):PC_70_BM BHJ solar cell based on the newly designed P-IDI-SVS donor polymer. The additional additive introduced for the additive control was CN, and the device produced a highest PCE of 3.38% using CB–CN3%. This resulted in an improved surface morphology, forming a much more uniform, well-aligned network and resulting in efficient charge transport. AFM, PL, TCSPC and Raman Spectroscopy measurements were performed to demonstrate the engineering effects of these solvent.

## Supplementary information

**Additional file 1: Fig. S1.** Solubility test images of P-IDI-SVS from different Soxhlet solvents of CB, CF, and THF at 80 °C without magnetic bars (donor only in CB solvent). **Fig. S2.** UV-vis absorption spectra of P-IDI-SVS (THF) with solution, film and annealed Film. **Fig. S3.** Cyclic voltammetry measurements of P-IDI-SVS. **Table S1.** Performance of different Soxhlet method of P-IDI–SVS:PC_70_BM (CB, CF, and THF) and BHJ devices processed from solutions with CB, CB–DIO3% and CB–CN3%.** Table S2.** Fitting parameter of TCSPC analysis with additive CN.

## Data Availability

The authors have no data to share since all data are shown in the submitted manuscript.

## Abbreviations

P-IDI-SVS: poly(indoloindole-selenophene vinylene selenophene)
OSCs: organic solar cells
BHJ: bulk heterojunction
CB: chlorobenzene
CF: chloroform
THF: tetrahydrofuran
AFM: atomic force microscope
PL: photoluminescence
TCSPC: time-correlated single-photon counting
HTL: hole transport layer
GBL: γ-butyrolactone
DMSO: dimethylsulfoxide
DSC: differential scanning calorimetry
TGA: thermogravimetric analyser
ITO: indium tin oxide
IPA: isopropyl alcohol
CN: 1-chloronaphthalene
DIO: diiodooctane
EQE: external quantum efficiency
