# Supplementary material for: Selective Soxhlets extraction to enhance solubility of newly-synthesized poly(indoloindole-selenophene vinylene selenophene) donor for photovoltaic applications
Source: Nano Converg. 2020 Mar 10;7:9. doi: 10.1186/s40580-020-0219-9 (PMC7062981; doi:10.1186/s40580-020-0219-9)
Supplement: Supplementary file 1 — Additional file 1: Fig. S1. Solubility test images of P-IDI-SVS from different Soxhlet solvents of CB, CF, and THF at 80 °C without magnetic bars (donor only in CB solvent). Fig. S2. UV-vis absorption spectra of P-IDI-SVS (THF) with solution, film and annealed Film. Fig. S3. Cyclic voltammetry measurements of P-IDI-SVS. Table S1. Performance of different Soxhlet method of P-IDI–SVS:PC70BM (CB, CF, and THF) and BHJ devices processed from solutions with CB, CB–DIO3% and CB–CN3%. Table S2. Fitting parameter of TCSPC analysis with additive CN. [file 40580_2020_219_MOESM1_ESM.docx]

Additional Information

**Selective Soxhlets Extraction to Enhance Solubility of Newly-Synthesized Poly(Indoloindole-selenophene vinylene selenophene) Donor for Photovoltaic Applications**

Jihyun Lim ^a,+^, Na Yeong Kim ^b,+^, Woongsik Jang ^a^, Un Su An ^b^, Aung Ko Ko Kyaw ^c^, Yun-Hi Kim^b,^*, and Dong Hwan Wang^a,^*

^a^School of Integrative Engineering, Chung-Ang University, 84 Heukseok-Ro, Dongjak-gu, Seoul 156-756, Republic of Korea

^b^Department of Chemistry and RINS, Gyeongsang National University, Jin-ju, 660-701, Republic of Korea

^c^Department of Electrical and Electronic Engineering, Southern University of Science and Technology Shenzhen 518055, P.R. China

*Corresponding authors, E-mail addresses: ykim@gnu.ac.kr (Prof. Y.H. Kim) and king0401@cau.ac.kr (Prof. D.H. Wang)

^+^Both authors contributed equally to this work.

Keywords: indoloindole, donor polymer, solar cell, Soxhlet, additive, organic solar cells


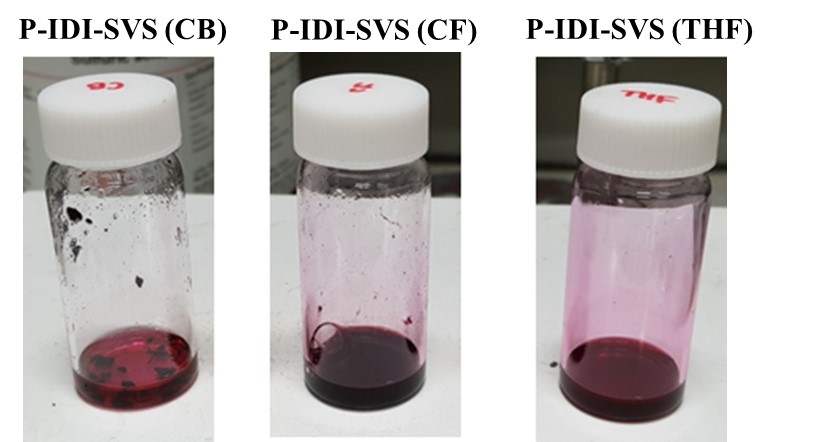


**Fig. S1**. Solubility test images of P-IDI-SVS from different Soxhlet solvents of CB, CF, and THF at 80 °C without magnetic bars (donor only in CB solvent).

**Fig. S2**. UV-vis absorption spectra of P-IDI-SVS (THF) with solution, film and annealed Film.

**Fig. S3**. Cyclic voltammetry measurements of P-IDI-SVS.

| Active layer | V_OC_  [V] | J_SC_  [mA/cm^2^] | FF  [%] | PCE  [%] |
| --- | --- | --- | --- | --- |
| P-IDI-SVS(THF) in CB | 0.583 | 5.37 | 57 | 1.77 |
| P-IDI-SVS(CB) in CB-DIO3% | 0.516 | 7.42 | 55.9 | 2.14 |
| P-IDI-SVS(CF) in CB-DIO3% | 0.596 | 6.72 | 56.1 | 2.25 |
| P-IDI-SVS(THF) in CB-DIO3% | 0.564 | 9.97 | 56 | 3.15 |
| P-IDI-SVS(THF) in CB-CN3% | 0.57 | 9.68 | 61 | 3.38 |

**Table S1**. Performance of different Soxhlet method of P-IDI–SVS:PC_70_BM (CB, CF, and THF) and BHJ devices processed from solutions with CB, CB-DIO3% and CB-CN3%.

**Table. S2**. Fitting parameter of TCSPC analysis with additive CN.

|  | A_1_  (%) | τ_1_  (ns) | A_2_  (%) | τ_2_  (ns) | τ_avg_  (ns) |
| --- | --- | --- | --- | --- | --- |
| CB | 99.6 | 0.492 | 0.4 | 3.108 | 0.50 |
| CB-CN3% | 99.9 | 0.349 | 0.1 | 2.471 | 0.35 |
